# Highly electromagnetic transparent ceramic composite made of boron nitride nanotubes and silicon oxynitride via perhydropolysilazane infiltration method

**DOI:** 10.1038/s41598-022-18563-4

**Published:** 2022-08-23

**Authors:** Ni Yang, Shaofan Xu, Chengying Xu

**Affiliations:** Department of Mechanical and Aerospace Engineering, NC State University, Raleigh, NC 27607 USA

**Keywords:** Engineering, Materials science, Physics

## Abstract

With the rapid development of electromagnetic (EM) wave circuit devices, high-performance wave-transparent materials with various functions have attracted great attention. Ceramic material is a promising candidate to be applied in harsh environments because of its chemical and corrosion resistance. In this work, a polymer-derived route was adopted to synthesize ceramic composite at room temperature. The composite is made of perhydropolysilazane-derived SiON ceramic and reinforced with boron nitride nanotubes (BNNTs) sheets. With the addition of SiON ceramic materials, the resultant sample showed an excellent hydrophobicity with a contact angle of 135–146.9°. More importantly, superior thermal stability at 1600 °C in the oxygen-containing atmosphere was observed for the fabricated SiON/BNNTs sample, without any shape change. The electromagnetic transparency of the SiON/BNNTs was studied through the waveguide method. The prepared SiON/BNNTs sample has an average real permittivity between 1.52 and 1.55 and an average loss tangent value in the range of 0.0074–0.0266, at the frequency range of 26.5–40 GHz. The effect of thickness on the wave transparency of SiON/BNNTs samples is also discussed. To summarize the aforementioned superior characterization and measurement results, the presented SiON/BNNTs material system has a great potential to be used as EM transparent materials in harsh conditions.

## Introduction

Wave-transparent materials have attracted prime attention over the past few decades as this type of material is of vital importance to the manufacturing of antenna housings and to protect the radar antenna system from the surrounding medium^1^. In general, qualified wave-transparent materials possess two characteristics, low dielectric permittivity (ε < 4) and low loss tangent (tanδ: 10^−2^–10^−3^)^2,3^, to reduce energy consumption. Wave-transparent polymer and ceramic materials are two main categories that are widely used in radio systems of hypersonic aircraft, reentry vehicles, high-speed missiles, and other similar devices^4,5^. Compared to polymeric composites, the wave-transparent ceramic materials^2,6^ have additional unique advantages with high melting points, abrasion resistance, atmospheric corrosion resistance, and more stability in harsh environments. For example, Zinc Sulfide (ZnS)^7^ is one of the most common long-wave infrared antenna window materials since the 1960s, and its excellent performances in mechanical/thermal/manufacturing properties have been extensively investigated by others. However, the harsh demands from the severe workplace and the requirement for weight reduction pushed the focal point into a challenging area of lightweight and wave-transparent performance, which encompasses the desirable traits of both polymers and ceramics.

Boron nitride nanotubes (BNNTs), are cylinders with sub-micrometer diameters and micrometer lengths. They have attractive properties exhibited by the combination of a low dielectric constant and a high modulus of elasticity^8–10^. BNNTs have been applied as one kind of reinforcement material to make ceramic composites with exceptional thermal conductivity and dielectric constant^11,12^. BNNTs are a low-k dielectric material with a relative dielectric constant ranging from 1.0 to 1.1 (50 Hz–2 MHz)^8^, and they are promising for mechanical applications due to the high modulus. For example, BNNTs reportedly have an excellent Young’s modulus (estimated up to 1.22 ± 0.24 TPa)^13^, varying on the nanotube diameter and thickness^14^. Thus, BNNTs can be a potential candidate for use in high-temperature wave-transparent applications because of their low dielectric constant and loss tangent, excellent ultra-lightweight structure, and high melting point. However, based on the potential applications of wave-transparent materials in high-speed missiles, the remarkably high thermal conductivity (21.39 W/mK at 25 wt% BNNTs)^15^ may limit its further applicability in this field. Glass–ceramic^16^, a novel polycrystalline solid material, is made of microcrystalline and amorphous phases, and it has also received increased interest recently. Silicon oxynitride (SiON) belongs to the glass–ceramic family, and its ultra-low thermal conductivity (1.1–1.4 W/mK) and relative dielectric constant (3.7–3.9)^17^ may compensate for the shortcomings that BNNTs possess. Specifically, the SiON-coated BNNTs can be the basis for revolutionary new materials and processes, and this first-mentioned novel composite will shed some light on wave-transparent materials.

Typically, SiON powders can be synthesized through the reaction between silica powders and ammonia^18^. However, the amount of nitrogen incorporation during the reaction cannot be controlled accurately, and thus the quality of the resulting SiON product is unstable. Moreover, these formation methods are costly and complex. To solve the conventional and costly formation methods of SiON, the polymer-derived ceramics (PDCs) route^19^ can be applied as it provides a novel technique that allows the thermal processing of the ceramic to be carried out at a relatively low temperature or even at room temperature. More importantly, the material denoted as PDCs allows the tailoring of polymeric precursors to produce elaborate shapes and alter their phase compositions^20^. For example, a recent work^21^ stated that PDCs route allows tailoring the electrocatalytic performance to elaborate complex ultrathin silicon and carbon (Si–C) based ceramic systems with 2D reduced graphene oxide (rGO), which cannot be obtained by a conventional method. Extensive studies have shown the promising potential for the fabrication of oxide or non-oxide ceramics, derived from specific polymer precursors. As reported in our earlier work^22^, one kind of preceramic polymer called perhydropolysilazane (PHPS), has a repeated structure of [–H_2_Si–NH–]_n_ and can be converted into SiON or SiO_2_ under Nitrogen and air conditions, respectively. The molar ratio of x and y in SiO_x_N_y_ is dependent on the annealing temperature and conditions^23^. Different from other PDC materials with an annealing temperature over 1000 °C, PHPS has a unique ability to convert into amorphous SiON ceramics at or close to room temperature during the liquid-to-ceramic transformation. To the best of our knowledge, no such material with superior performance as a wave-transparent material has been proposed to date.

In this paper, we proposed and studied an ultra-lightweight electromagnetic (EM)-transparent ceramic material composed of SiON coated BNNTs, based on the PDCs route at room temperature. This single layer of thermal-resistant ceramic (~ 0.3 mm) is over 90% transparent to radio waves. The highest its transparency reaches is 97%. The BNNTs mat consists of a number of nanotubes that are held together by van der Waals forces. A certain amount of PHPS-derived SiON is presented to hold the nanotubes together at their junctions and remove air voids. The effects of the polymer-derived SiON addition on the resulting phase/structural evolution, thermal behavior, and EM transparency of SiON/BNNTs ceramic are systematically investigated.

## Experimental part

### Materials

The commercially available boron nitride nanotubes (BNNTs) puffballs, provided by BNNT, LLC (SP10-R, Newport News, VA) involve the HTP method during the manufacturing process. A proprietary purification method was used by the vendor to remove almost all of the elemental boron to produce this refined product with more than 99% boron nitride. Polysilazane (NN120-20 (A), durXtreme GmbH, Germany) was obtained in the format of a 20 wt.% solution of perhydropolysilazane (PHPS) in di-n-butyl ether.

### Synthesis of BNNTs/SiON composites

To prepare the refined mat, a BNNT SP10-R refined puffball was selected and placed between two weighing papers (nitrogen-free, 4*4 inch, LAB SAFETY SUPPLY™) with the edges stapled. Then, the abovementioned materials were placed between two steel plates and a uniaxial pressure was applied by a benchtop press (Model 4386, CARVER^Ⓡ^, USA) until a BNNTs sheet with the thickness of 0.2 mm formed. Subsequently, the BNNTs sheets were completely immersed in the PHPS solution. After the infiltration was complete, the wet sample was squeezed out and the excess PHPS solution on the surface of the sample was removed using paper towels. The drying process took place overnight, and the filtration step was repeated 3 times to synthesize the SiON/BNNTs composite. The thickness of the final sample was around 0.3 mm.

### Characterization method

Thermal stability was analyzed using a Discovery DSC250 (TA Instruments, USA) under an air atmosphere. Pure BNNTs, PHPS-derived SiON, and SiON/BNNTs composite were weighed into Tzero aluminum pans (TA instruments) and measured in a heating cycle from room temperature to 950 °C at 10 °C/min, respectively. Further thermal analysis was conducted in a tube furnace (Carbolite Gero 30–3000 °C, USA) at 1000 °C in the air for up to 100 h.

The XRD analysis was measured with a multifunctional Rigaku SmartLab X-ray diffractometer (Rigaku Corporation, Tokyo, Japan) equipped with a rotating copper cathode in the Bragg‐Brentano configuration. Samples were scanned at a step size of 0.25° in a 2θ range of 10°–90°. High-temperature X-ray diffraction measurements were carried out using PANalytical Empyrean diffractometer with Anton Paar HTK 1200. The sample was heated from 25 to 1000 °C with a ramp of 2 °C/min and a dwell time of 60 min.

The micro/nanostructures of the composite sample were characterized by a field-emission scanning electron microscope (FE-SEM, FEI Verios 460L). The hydrophilic or hydrophobic characteristics of pure BNNTs and SiON/BNNTs composites were evaluated via the contact angle measurement (Ramé-hart Model 260 Contact Angle Goniometer), measured using the tangent line between a drop of water and the sample surface.

The S-parameters and permittivity were measured using the waveguide method. The measurement setup consisted of a vector network analyzer (Keysight, N5225A PNA, 10 MHz–50 GHz), coaxial cable, waveguide cavity, calibration kit (Keysight, R11644A, 26.5–40 GHz), and sample holder. The scattering parameters (S-parameters) were directly measured and recorded by PNA, and the permittivity was calculated according to Nicolson–Ross–Weir (NRW) algorithm. The relative complex permittivity of samples with dimensions of 7.112 mm × 3.556 mm × 3.556 mm was measured within Ka-band frequency (26.5–40 GHz).

We used Origin 2019 (64-bit) with the version of 9.6.0.172 (Academic) to complete Figs. 1, 4b,c, 5b, and 6, 7, 8, 9 and 10. This Origin is owned by North Carolina State University and retrieved from www.originlab.com.

**Figure 1:**
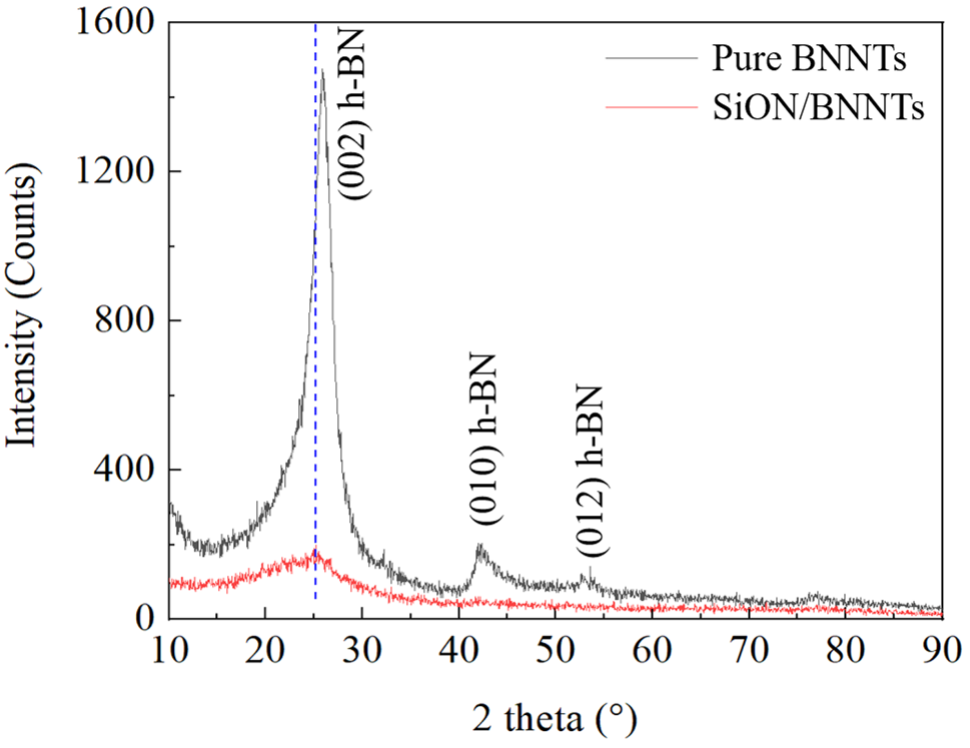
Room-temperature Bragg–Brentano geometry X-ray diffraction (XRD) patterns of pure BNNTs and SiON/BNNTs. (This figure is completed using Origin 2019 (64-bit) with the version of 9.6.0.172 (Academic), which is retrieved from https://www.originlab.com).

## Results and discussions

### Structural evolution

The XRD patterns of BNNTs and SiON/BNNTs composite were presented in Fig. 1. For the pure BNNTs sample, all reflection peaks were located at 25.79°, 42.13°, and 53.20°, with the corresponding (002), (010), and (012) crystallographic planes. They showed the dominant BN phases which were mainly composed of hexagonal-BN with lattice constants of a, b = 2.498 Å and c = 6.636 Å (reference code: 98-012-3398)^24^. Only the (002) peak of BNNTs was visible in the sample of SiON/BNNTs, which was due to the amorphous structure of PHPS-derived SiON at room temperature. The structure of the converted SiON was the combination of amorphous SiO_2_ and partially uncondensed Si–N, Si–OH, and O–H^25^. The amorphous nature of this material was illustrated in the work of Funayama et. al^26^, where one broad peak at less than 20° was detected and attributed to the amorphous structure. Compared to the (002) position of pristine BNNTs at 25.8°, the corresponding peak in SiON/BNNTs sample was slightly shifted to lower than 2θ at 23°. One was resulting from the amorphous nature of the SiON addition, and another was because of the slight expansion of the interplanar spacing in the nanometric morphology of BNNTs.

**Figure 2:**
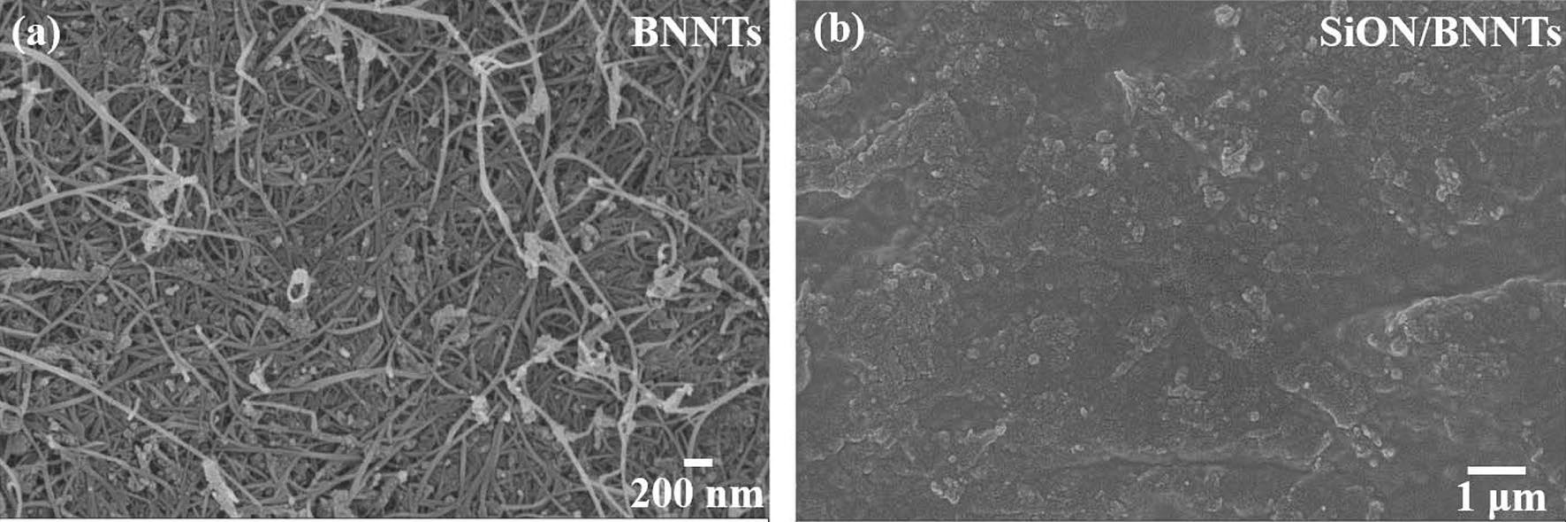
Structural characterization: SEM surface and cross-section images of (**a**) BNNTs and (**b**) SiON/BNNTs composites.

Understanding the micro- and nanostructures is also important in this work to fully investigate the effect of polymer-derived SiON ceramic on the properties of BNNTs. Figure 2a showed the high density of BNNTs with diameters of 30–50 nm and a very high aspect ratio. The existence of dots dispersed inside the matrix was due to the remaining boron during BNNT fabrication^27^. Figure 2b illustrates the surface changes after the infiltration of SiON ceramic between the BNNTs. It is evident that the surface was dense and flat, which was different from pure BNNTs. The empty gaps between the BNNTs were filled by SiON successfully, via the polymer-derived route in this study. This phenomenon reduced the influence of porosity on the S-parameters and permittivity measurements in “Wave-transparent properties”.

**Figure 3:**
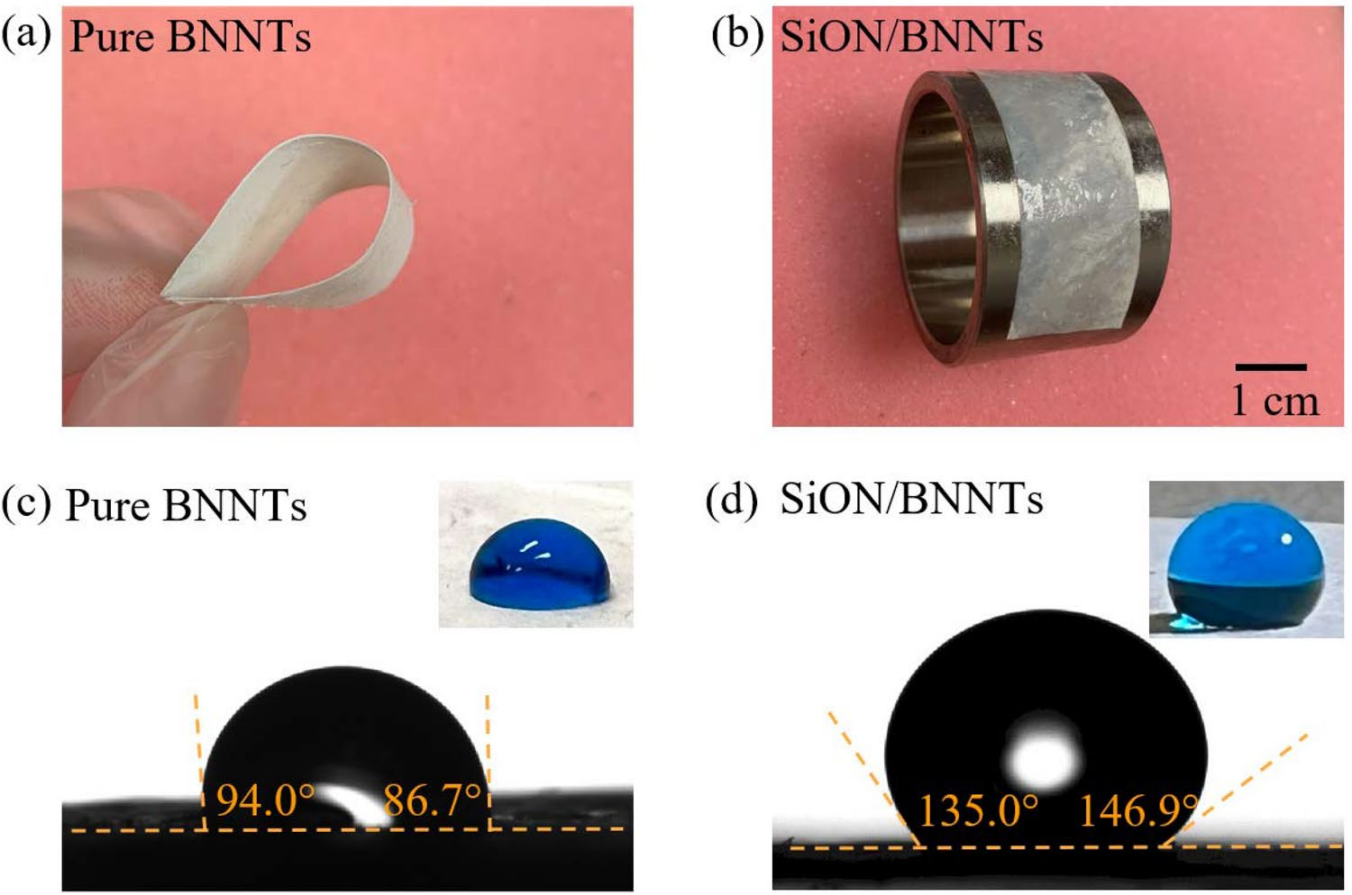
(**a**, **b**) Flexibility exhibition and (**c**, **d**) contact angles of pure BNNTs and SiON/BNNTs materials, respectively.

To be applied as a radome material, the material must protect the radar antenna system from the surrounding medium while remaining formable; thus, such material must be flexible. The pure BNNTs and SiON/BNNTs materials are available in thin, lightweight ceramics for flexibility in radome design and layup, and various curved shapes can be achieved. As seen in Fig. 3a, the prepared BNNTs sheet was highly flexible and foldable, which could easily be recovered to the original morphology shortly after release. Due to its highly flexible nature, the PHPS infiltrated BNNTs film easily rolled up on a curved metal surface (Fig. 3b), illustrating potential applicability as a radome material. The wettability of the surface of the material also determines whether it can resist the effects of rain on its service life. In this study, wettability was characterized by the contact angle of water with the solid surface from pure BNNTs and SiON/BNNTs materials in Fig. 3c,d. A smaller contact angle indicates the greater wettability of the materials. The contact angles on pure BNNTs and SiON/BNNTs were 86.7–94.0° and 135–146.9°, respectively. These results revealed that the pure BNNTs were inbetween hydrophilicity and hydrophobicity, while the SiON coated BNNTs showed significant hydrophobicity. This conclusion shows that the coating derived from PHPS is a low surface energy material that can be applied on nanostructures of BNNTs to reduce surface energy. The addition of SiON coatings provides hydrophobic surfaces that play an important role in reducing the possible damage from rain.

### Thermal stability

**Figure 4:**
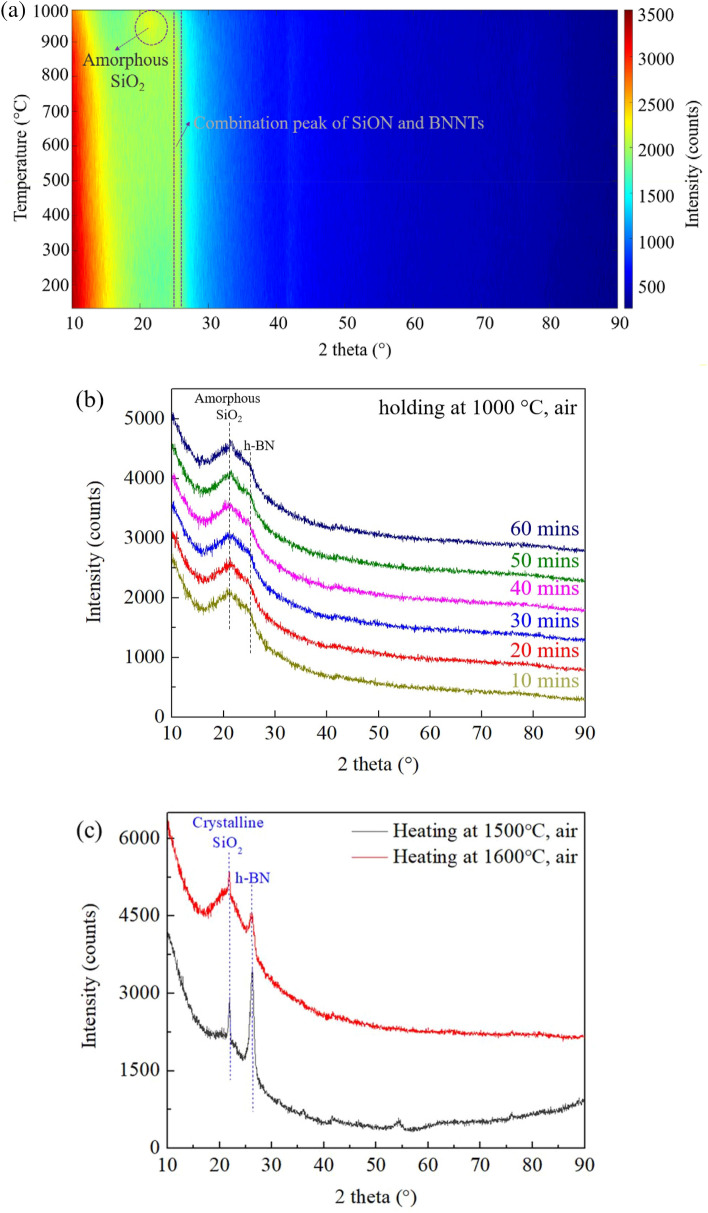
Series of XRD patterns were recorded for the SiON/BNNTs composite from (**a**) room temperature to 1000 °C with the heating rate of 2 °C/min (air), (**b**) holding at 1000 °C for 1 h (air), and (**c**) heating at 1500 and 1600 °C for 1 h (air), respectively. ((**b**, **c**) are completed using Origin 2019 (64-bit) with the version of 9.6.0.172 (Academic), which is retrieved from https://www.originlab.com).

To further investigate the thermal protection provided by the addition of PHPS-derived SiON, HT-XRD can provide useful information on its thermal behavior in harsh environments. The numbered series of in situ high temperature (HT)-XRD scans in Fig. 4 highlight the major advantage of the PHPS-derived SiON/BNNTs compared to the pure BNNTs. As it can be seen, the small peak at 24.8° in Fig. 4a was the combination of SiON and BNNTs existences, which was consistent with the results in Fig. 1. With the heat treatment from 25 to 1000 °C, this peak was considered stable, but a minor change was detected at 900 °C around 23°. These broad diffraction peaks located at 23° were amorphous SiO_2_, which has been reported in our earlier study^28^. PHPS contains extensive Si–H and Si–N groups, and the oxidation and hydrolysis of these Si–H groups can form Si–OH groups to produce amorphous SiON(H) ceramic at room temperature. It explains the broad peak between 20° and 30° of SiON/BNNTs samples in Fig. 1. As the temperature goes up, elemental O and N can gradually be released from the chemical structure, and N can be almost eliminated at 800–900 °C^20^. The plethora of the Si–OH bonds subsequently condense to synthesize numerous Si–O–Si bonds to form a SiO_2_-rich phase and that is the reason it is shown at HT-XRD in Fig. 4a–c. The abovementioned processes are shown in Eqs. (1)–(4):

1 $${\text{Si}} - {\text{H}} + {\text{O}} - {\text{O}} \to {\text{Si}} - {\text{OH}}$$

2 $${\text{Si}} - {\text{H}} + {\text{H}} - {\text{O}} - {\text{H}} \to {\text{Si}} - {\text{OH}}$$

3 $${\text{Si}} - {\text{N}} + {\text{Si}} - {\text{OH}} \to {\text{Si}} - {\text{O}} - {\text{N}} - {\text{H}}$$

4 $${\text{Si}} - {\text{OH}} + {\text{Si}} - {\text{NH}} \to {\text{Si}} - {\text{O}} - {\text{Si}} + {\text{NH}}_{{3}} \uparrow$$

For Fig. 4b, these XRD patterns are measured every 10 min at the dwelling time at 1000 °C in the air, and it is evident that the SiON coating on the BNNTs has converted into SiO_2_ coating at higher temperatures. This will offer the best thermal stability and insulation at the working environment. The sample was further heated to 1500 °C and 1600 °C under air (Fig. 4c). The test at 1700 °C was not selected due to the melting point of PHPS-derived ceramic (~ 1710 °C). As it can be seen in the sharp peak in XRD, the amorphous nature of SiO_2_ caused it to crystallize at 1500 °C and 1600 °C. The appearance of the h-BN peak still demonstrated the sample was strongly protected by the SiO_2_ covering in the harsh condition. This transformation to SiO_2_ signifies that the proposed SiON/BNNTs composites possess excellent thermal-resistance performance even at 1600 °C in the oxygen-containing atmosphere.

**Figure 5:**
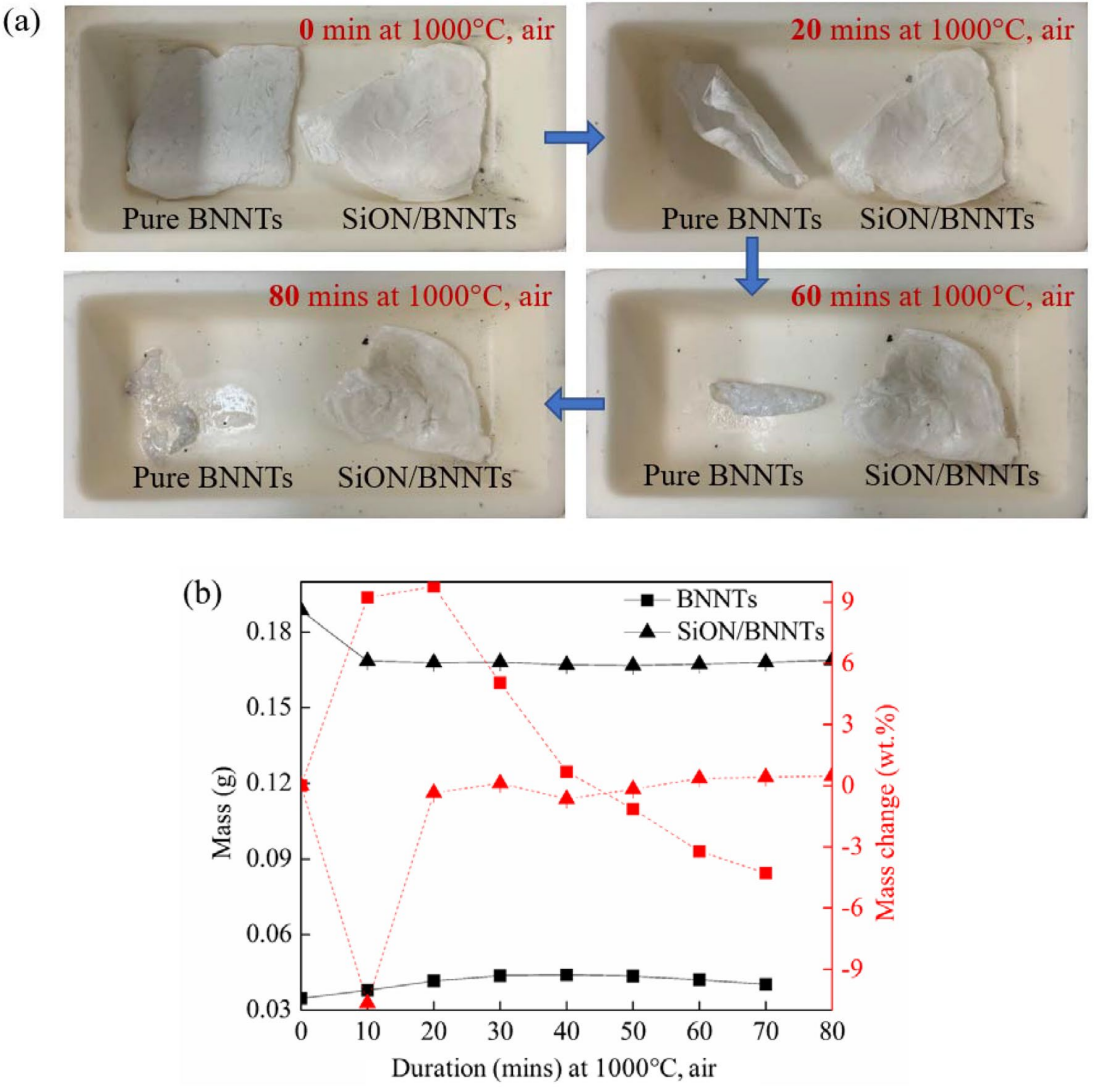
(**a**) Sample change and (**b**) mass changes of pristine BNNTs and PHPS-derived ceramic/BNNTs samples with different dwelling times at 1000 °C. ((**b**) is completed using Origin 2019 (64-bit) with the version of 9.6.0.172 (Academic), which is retrieved from https://www.originlab.com).

To better display the superior protection from PHPS-derived ceramic coating on the bulk BNNTs, the SiON/BNNTs composite and its control group-pure BNNTs were both placed in an Alumina crucible and tested at 1000 °C thermal treatment in air. From the changes in the samples’ appearance in Fig. 5a, it is noted that the pristine BNNTs began to curl and shrink after only 20 min at 1000 °C, while the SiO(N)/BNNTs composite maintained their shape regardless of its time in high temperatures. When the heat preservation experiment was carried out for the initial 60 min, the BNNTs began to “melt”. This melting is because the BNNTs can only maintain their oxidation resistance up to 800–900 °C^29^, and they can be partly transformed to boron oxide at 1000 °C^30^. Based on these findings, the SiON ceramic coating is an effective and simple method to make BNNTs more desirable for numerous applications in harsh conditions.

Figure 5b depicts the mass loss within 0–80 min after high-temperature oxidation treatment at 1000 °C. The weight of BNNTs over 80 min was unattainable as the sample melted and stuck to the container. A slight weight gain during the initial 40 min was detected for pure BNNTs, due to the oxidation of BN^31^ and boron^32^. The formation of B_2_O_3_ results in a rapid weight gain and the relevant chemical reaction following Eqs. (5) and (6):

5 $${\text{BN}} + {\text{O}}_{{2}} \to {\text{B}}_{{2}} {\text{O}}_{{3}} + {\text{N}}_{{2}}$$

6 $${\text{B}} + {\text{O}}_{{2}} \to {\text{B}}_{{2}} {\text{O}}_{{3}}$$

The subsequent weight loss may be due to the missing residual melt when weighing. The SiON/BNNTs composite displayed a major weight loss of 10.66 wt% over the first 10 min which can be attributed to the evaporation of the atmospheric moisture and the loss of N–H and Si–H parts^20^. After this initial weight loss, the weight trace from the SiON/BNNTs was relatively stable, indicating that the existence of SiON improved the oxidation behavior and thermal stability of pure BNNTs.

**Figure 6:**
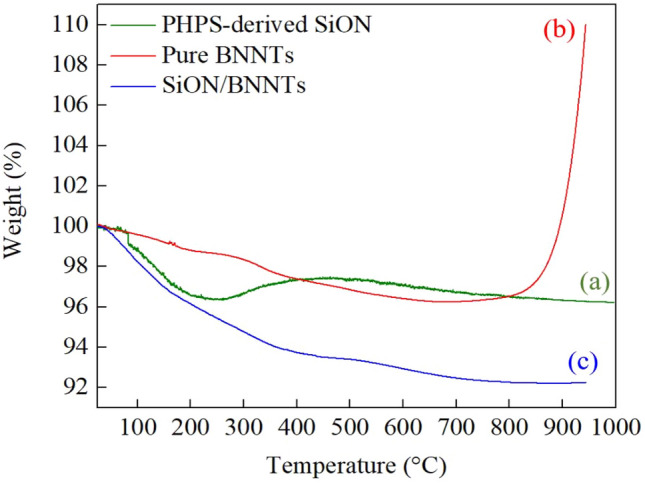
TGA curve of (**a**) PHPS-derived SiON, (**b**) pure BNNTs, and (**c**) PHPS-derived SiON/BNNTs samples. (This figure is completed using Origin 2019 (64-bit) with the version of 9.6.0.172 (Academic), which is retrieved from https://www.originlab.com).

The changes in mass as a function of temperature were characterized by the TGA technique, and the corresponding results were shown in Fig. 6. The results align with the discussion from Figs. 4 and 5. During the heating step of 25–200 °C, the occurrence of quick weight losses (~ 4 wt%) for PHPS-derived SiON and SiON/BNNTs were due to the evaporation of the remaining organic solvent and the loss of N–H and S–H species as explained before. For pristine BNNTs, the removal of moisture resulted in the weight loss of 1.33 wt% during this phase. Different from the continuous weight loss for BNNTs and SiON/BNNTs samples after 250 °C, a weight gain of ∼ 1.06% from 250 to 450 °C was observed for the PHPS-derived SiON. This weight gain suggests the oxidation of Si–NH as shown in Eq. (3). The main condensation of the Si–OH bonds caused the weight loss at more than 450 °C. It has been reported that the silanol groups are highly likely to generate Si–O–Si bonds through the self-condensation process^33^. In Fig. 6b, the weight gain of pure BNNTs resulted from the oxidation of boron, which was also explained in Eq. (5). Overall, the studied SiON/BNNTs composite is thermally stable up to 1000 °C in the air with > 92 wt% mass retention without shape changes. The addition of SiON greatly improved the heat resistance of BNNTs, especially in a sustained 1000 °C environment.

### Wave-transparent properties

**Figure 7:**
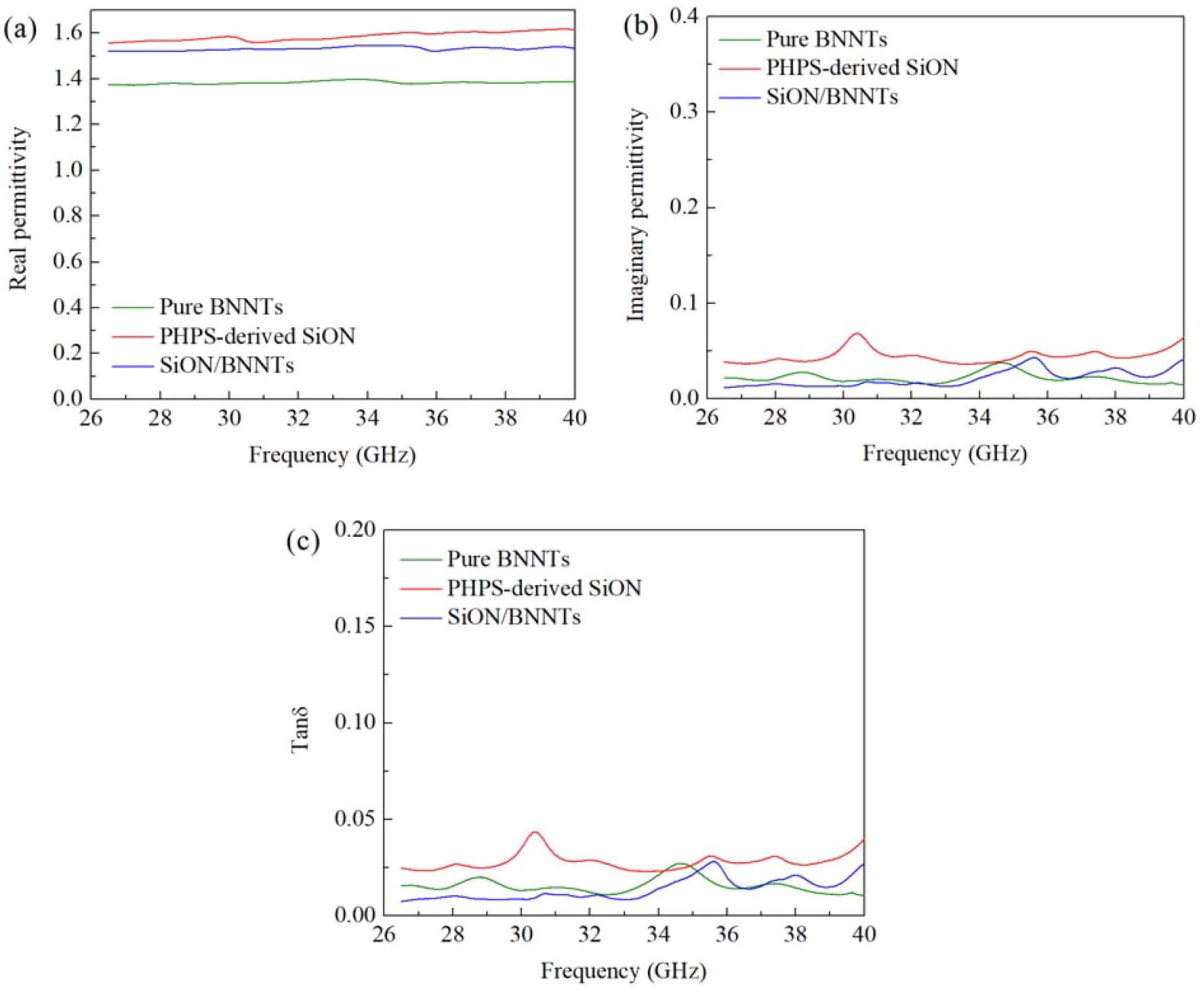
Complex permittivity: (**a**) real permittivity and (**b**) imaginary permittivity; and (**c**) loss tangent of pure BNNTs, PHPS-derived SiON, SiON/BNNTs samples (thickness ~ 6 mm) at frequencies of 26.5–40 GHz measured at room temperature, showing good electromagnetic wave-transparent performance. (This figure is completed using Origin 2019 (64-bit) with the version of 9.6.0.172 (Academic), which is retrieved from https://www.originlab.com).

One of the most attractive properties of the PHPS-derived SiON ceramics is the low complex permittivity and loss tangent. The permittivity testing results are depicted in Fig. 7. All specimens showed relatively low real permittivity (ε′ < 1.62) and imaginary permittivity (ε″ < 0.07), which meets the requirement for radome applications. The value of the ε′ for pure BNNTs was ~ 1.38 in the whole frequency range. PHPS-derived SiON ceramics illustrated higher values of ε′ between 1.55 and 1.62, relative to the pure BNNTs. This phenomenon is due to the higher polarization capability of PHPS-derived SiON. Also, the infiltration of SiON removed all the pores and created a greater density in the resultant materials. The nature of dipole polarization results in the value of the real permittivity (ε′) of SiON > the value of SiON/BNNTs > the value of BNNTs. If the external field frequency is low, the polarization in the medium can follow the change of the external field, which means there is no polarization loss. In the condition where the external field frequency increases, the required polarization stability time will be longer than the period of the external field’s shift, and the polarization loss will be introduced.

The analysis of the equation can begin with the Debye equation:

7 $$\varepsilon \left(\omega \right)={\varepsilon }_{\infty }+\frac{{\varepsilon }_{s}-{\varepsilon }_{\infty }}{1+j\omega \tau }=\frac{{\varepsilon }_{s}-j\omega \tau {\varepsilon }_{\infty }+j\omega \tau {\varepsilon }_{s}+{\omega }^{2}{\tau }^{2}{\varepsilon }_{\infty }}{1+{(\omega \tau )}^{2}}$$

8 $${\varepsilon }^{^{\prime}}=\frac{{\varepsilon }_{s}-{\varepsilon }_{\infty }}{1+{(\omega \tau )}^{2}}+{\varepsilon }_{\infty }$$

9 $${\varepsilon }^{^{\prime\prime} }=\frac{{\varepsilon }_{s}-{\varepsilon }_{\infty }}{1+{(\omega \tau )}^{2}}\omega \tau$$

where *ε*′ is the real permittivity, *ε*″ is the imaginary permittivity, $\omega$ is the angular frequency, $\tau$ is the relaxation time, ${\varepsilon }_{s}$ is the static permittivity (at low frequency), and ${\varepsilon }_{\infty }$ it the permittivity at extremely high frequency. It can be seen from the formulas that as the frequency increases, the value of ${\varepsilon }^{^{\prime}}$ decreases. However, as the frequency increases to some extent, the real permittivity will be almost constant within the frequency range. In the calculation of imaginary permittivity for a material, the conduction loss can also be found. Thus, the equation of the imaginary permittivity is updated to:

10 $${\varepsilon }^{^{\prime\prime} }=\frac{{\varepsilon }_{s}-{\varepsilon }_{\infty }}{1+{(\omega \tau )}^{2}}\omega \tau +\frac{\sigma }{\omega {\varepsilon }_{0}}$$

where $\sigma$ is the electrical conductivity of the material. This conduction normally occurs in the microwave region. Figure 7b shows the variation in the imaginary dielectric constant at different frequencies, and it shows that the BNNTs act as excellent electrical insulators. Overall, the value of ε″ of the pure BNNTs corresponded closely to that of SiON/BNNTs, with a range of 0.01–0.04. They both possess a lower value of electrical conductivity due to the limited electron density based on the classical electron/Drude–Lorentz Equation (Eqs. 7–9).

**Figure 8:**
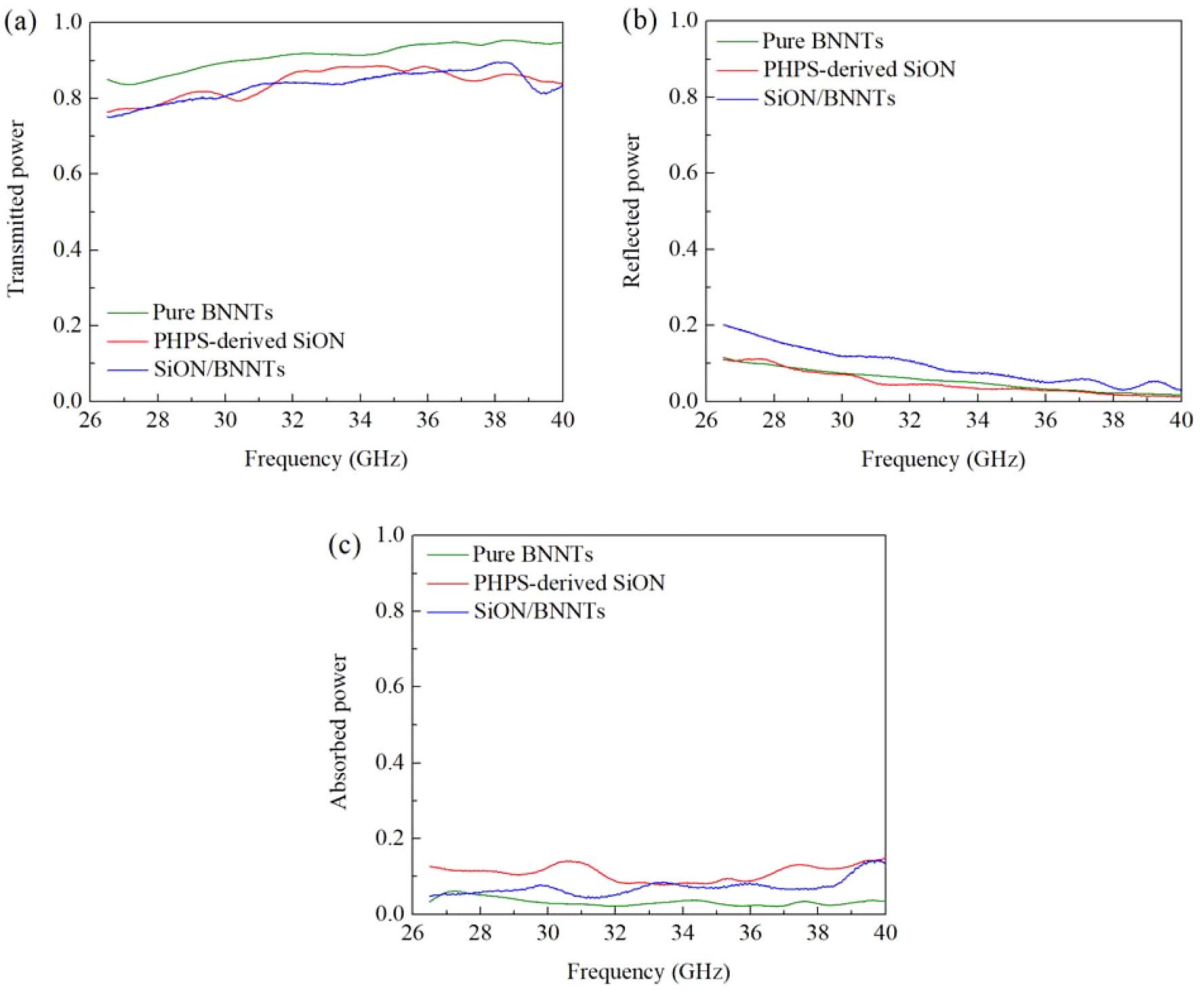
The (**a**) transmitted, (**b**) reflected, and (**c**) absorbed powers in percentage versus frequency of pure BNNTs, PHPS-derived SiON, SiON/BNNTs samples (thickness ~ 3 mm) at frequencies of 26.5–40 GHz. (This figure is completed using Origin 2019 (64-bit) with the version of 9.6.0.172 (Academic), which is retrieved from https://www.originlab.com).

Scattering parameters (S parameters) can be used to comprehensively describe how energy propagates through an electrical network. In this study, the samples were measured in a rectangular waveguide with a frequency ranging from 26.5 to 40 GHz. The measured S parameters, as well as absorption power, are shown in Fig. 8. According to the law of the conservation of energy, the total sum of transmitted, reflected, and absorbed powers is 1. In Fig. 8a, the capability of transmission increased with increasing frequency. However, higher frequencies are more sensitive to reflection, and that is why the curves in reflection power were decreasing at higher frequencies. Overall, pure BNNTs showed the highest transmission exceeding 85% at 26.5 GHz and achieving values as high as 95% at 40 GHz. With this superior performance, the prepared SiON/BNNTs samples also exhibited excellent transmission results between 76% and 89% at 26.5–40 GHz. No obvious difference was observed between the SiON/BNNTs and PHPS-derived SiON samples. This absence of contrast may be attributed to the infiltration of the PHPS liquid. The PHPS liquid was fully infiltrated into the BNNTs sheet. Then, the SiON ceramic filled the gaps inside the BNNTs, and more so on the surface of BNNTs. The SiON coating affected the matching effect and gave a similar result for both SiON and SiON/BNNTs. A low permittivity value indicated the desired matching degree.

The relationship between S parameters and power can be described as below:

11 $${\text{S}}_{21}\text{ in dB}=10\times\log_{10}{({\frac{\text{Transmitted power}}{\text{Total input power}})}^{2}}=10\times\log_{10}{}{({\text{S}}_{21}\text{ in percentage})}^{2}$$

12 $${\text{S}}_{11}\text{ in dB}=10\times\log_{10}{({\frac{\text{Reflected power}}{\text{Total input power}})}^{2}}=10\times\log_{10}{}{({\text{S}}_{11}\text{ in percentage})}^{2}$$

13 $$\text{Absorbed power}=\text{Total input power}-\text{Reflected power}-\text{Transmitted power}$$

The reason why BNNTs have the highest transmitted power can be explained in the following equations:

14 $${R}_{L}=20log|\frac{{Z}_{in}-1}{{Z}_{in}+1}|$$

15 $${Z}_{in}=\sqrt{\frac{{\mu }_{r}}{{\varepsilon }_{r}}}$$

where ${R}_{L}$ is the return loss, ${\varepsilon }_{r}$ is the complex relative permittivity, and ${\mu }_{r}$ is the complex relative permeability (which equals 1 for non-magnetic material). Based on the equations above, a lower relative permittivity will result in a lower return loss. The pure BNNTs have the smallest relative permittivity, which results in the lowest reflected power. However, the pure PHPS-derived SiON also has a low reflected power. These results appear to contradict the result obtained from the equations above. This phenomenon can be explained as the equations shown below, in Eqs. (16) and (17)

16 $${P}_{a}={P}_{0}\cdot {(1-e}^{-2(\alpha +j\beta )d})$$

17 $$\alpha =\frac{\sqrt{2}\pi f}{c}\times \sqrt{\left({\mu }^{^{\prime\prime} }{\varepsilon }^{^{\prime\prime} }-{\mu }^{^{\prime}}{\varepsilon }^{^{\prime}}\right)+\sqrt{{({\mu }^{^{\prime}}{\varepsilon }^{^{\prime\prime} }+{\mu }^{^{\prime\prime} }{\varepsilon }^{^{\prime}})}^{2}+{({\mu }^{^{\prime\prime} }{\varepsilon }^{^{\prime\prime} }-{\mu }^{^{\prime}}{\varepsilon }^{^{\prime}})}^{2}}}$$

where *P* is the absorbed power, ${P}_{0}$ is the power entered the sample, *d* is the thickness of sample, $\alpha ,$ and $\beta$ are the real and imaginary parts of the propagation constant. More specifically, the higher $\alpha$ is, the more power absorbed by the sample and consumed in the form of heat. $\alpha$ is closely related to the imaginary part of relative permittivity—the higher the imaginary part of relative permittivity, the more power absorbed by the material. This is the reason why most power is absorbed by SiON instead of being reflected, as shown in Fig. 8.

**Figure 9:**
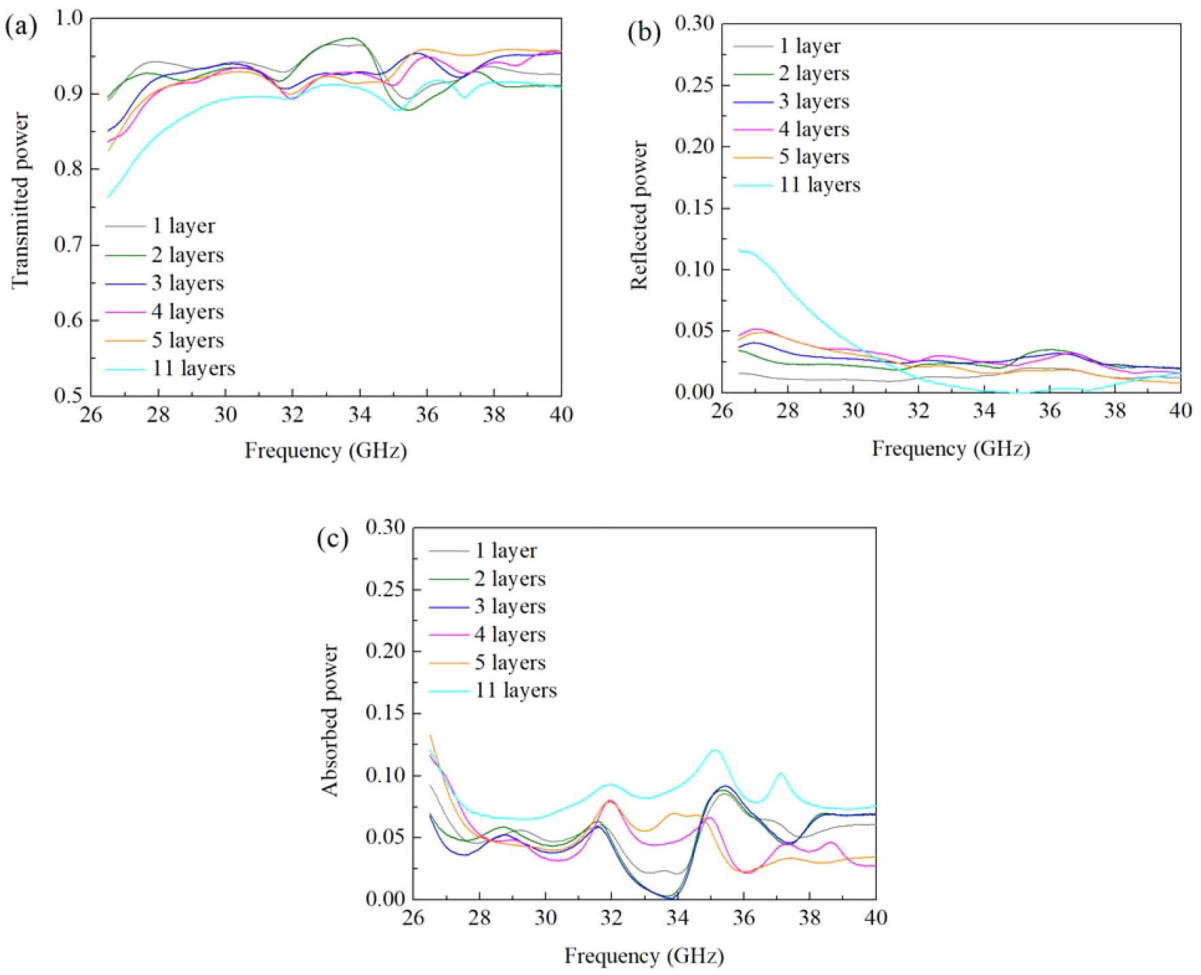
The (**a**) transmitted, (**b**) reflected, and (**c**) absorbed powers in percentage versus frequency of SiON/BNNTs samples at different thicknesses with the frequencies of 26.5–40 GHz. (This figure is completed using Origin 2019 (64-bit) with the version of 9.6.0.172 (Academic), which is retrieved from https://www.originlab.com).

Sample thickness also affects the wave-transparent properties. The increase in the material thickness *d* means that the propagation distance of the electromagnetic waves in the medium also increases. When the incident angle of the electromagnetic waves remains unchanged, the absorption loss increases while *d* also increases. Since the thickness of the material is increasing, the overall wave-transparent properties of the material decrease. Based on the results from Figs. 7 and 8, these three samples were both EM transparency materials, especially for pure BNNTs and SiON/BNNTs. To investigate the effect of sample thickness on the measured results, different layers of SiON/BNNTs samples were prepared with different thicknesses. In this experimental set, each layer had a thickness of 0.3 mm. The transmitted, reflected, and absorbed powers are shown in Fig. 9. It can be seen that the transmitted power decreased with the increasing thickness, and the transmitted power can be maintained at more than 90% for the entire frequency range studied. The reflected power, even with varying sample thickness, can still maintain a great impedance matching degree across the entire frequency range. No more than 5% power was reflected and lost based on the measurement results. This small value of the power reflection is due to the matching degree, which is primarily determined by the front surface of the material instead of sample thickness, as the thickness is much smaller than the wavelength.

As the SiON/BNNTs sample thickness increases, the transmitted power (S21) decreases mostly because of the increased absorption, as shown in Fig. 9a. The dissipated power increases exponentially with the increasing thickness, which can be shown in Eq. (15). However, as the thickness increases to a certain extent, the phase difference between the signal reflected from the first and second surfaces needs to be taken into consideration. As shown in Fig. 9b, with 11 layers of SiON/BNNTs and within the frequency range of 34–36 GHz, the reflection decreases to almost 0%. This minimal reflection occurs because the signals reflected from the top and bottom surfaces of the sample have a 180° phase difference and a similar magnitude, which cancel each other out.

**Figure 10:**
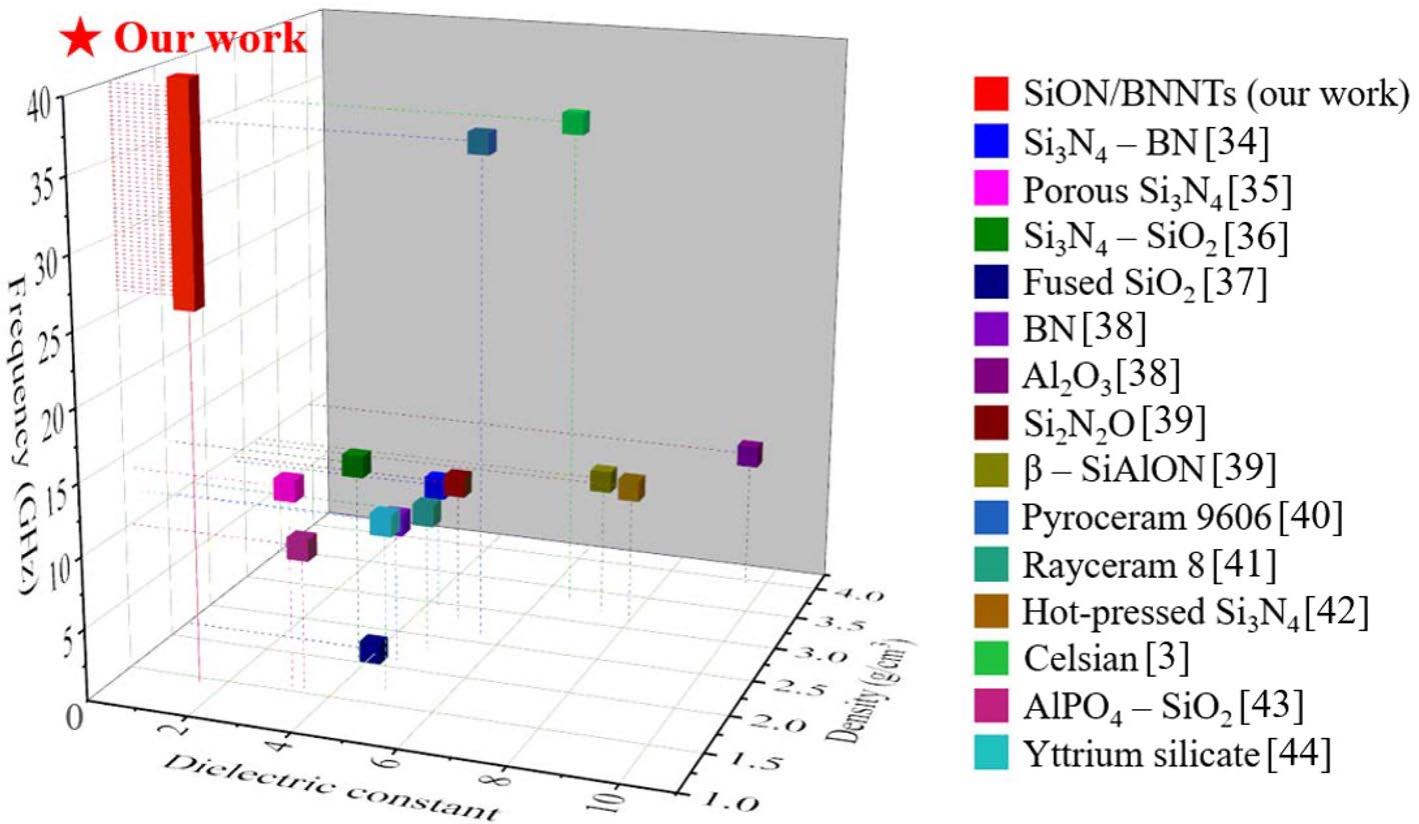
The dielectric constant and density of typical ceramic-based EM transparent composites from literature. (This figure is completed using Origin 2019 (64-bit) with the version of 9.6.0.172 (Academic), which is retrieved from https://www.originlab.com).

Specific dielectric properties of other EM transparent ceramics and composites from the literature^3,34–44^ are illustrated in Fig. 10. In comparison to other materials, our candidate showed a much lower dielectric constant (~ 1.51) and a lighter density (~ 1.5 g/cm^3^) at a wide frequency range (26.5–40 GHz). More importantly, the covering of PHPS-derived silica on the BNNTs provided a superior thermal resistance up to 1600 °C, which far exceeds the operating temperature of other products to the best of our knowledge. For example, Pyroceram 9606^40^, developed by Corning Glass, has a melting temperature of 1349 °C and a maximum operating temperature of approximately 1000 °C. Our flexible SiON/BNNTs bring the advantages of lighter weight, better EM transparent performance, as well as excellent thermal resistance. The improved operating temperature and the simplicity of the manufacturing process present a greater potential in scientific and technological fields. With these advancements, it can ignite the next generation of defense technology, such as satellite, sensor, Radar, and telecom communication systems. However, the cost and fragility due to extra thin thickness limit its use. This study will continue with the optimization of mechanical improvement (“Supplementary information”).

## Conclusion

Preparation of SiON thin film from perhydropolysilazane (PHPS) at room temperature has attracted great attention because it provides a new way to prepare ceramic materials while eliminating high-temperature processing steps. This paper reports on the creation of EM-transparent SiON/BNNTs composite based on perhydropolysilazane and pure BNNTs, through the PDCs route. The empty gaps between the BNNTs were successfully filled by SiON, and the surface of the flexible SiON/BNNTs ceramic showed significant hydrophobicity with a contact angle of 135–146.9°. Compared to that of pure BNNTs, the studied SiON/BNNTs composites possess excellent thermal-resistance performance at 1600 °C in the oxygen-containing atmosphere. More importantly, the prepared SiON/BNNTs samples exhibited excellent results on the electromagnetic (EM) transparency with an average real permittivity around 1.52–1.55 and an average loss tangent value in the range of 0.0074–0.0266, at 26.5–40 GHz. The transmitted power of SiON/BNNTs can achieve 0.90–0.97 with a thickness of 0.3 mm, and this superior wave-transparent property is maintained with increasing thickness. Such EM transparent material with superior performance will shed light on the applications of radome materials in harsh environment applications.

## Supplementary Information

Supplementary Information.

## Data Availability

The datasets used and/or analyzed during the current study available from the corresponding author C.X. (cheryl.xu@ncsu.edu) on reasonable request.
