# Supplementary material for: Highly electromagnetic transparent ceramic composite made of boron nitride nanotubes and silicon oxynitride via perhydropolysilazane infiltration method
Source: Sci Rep. 2022 Aug 23;12:14374. doi: 10.1038/s41598-022-18563-4 (PMC9399241; doi:10.1038/s41598-022-18563-4)
Supplement: Supplementary file 1 — Supplementary Information. [file 41598_2022_18563_MOESM1_ESM.docx]

**Highly Electromagnetic Transparent Ceramic Composite Made of Boron Nitride Nanotubes and Silicon Oxynitride via Perhydropolysilazane Infiltration Method**

Ni Yang, Shaofan Xu, Chengying Xu*

Department of Mechanical and Aerospace Engineering, NC State University

Raleigh, NC 27607

*Corresponding Author: Email: [cheryl.xu@ncsu.edu](mailto:cheryl.xu@ncsu.edu)

1. Structural comparison of BNNTs (loose, porous) and SiON/BNNTs (flat, dense)

It is obvious that the surface of the BNNTs/SiON sample is relatively flat and dense, no matter which scale bar is being used. However, BNNTs show the nature of the loose structure, with B particles dispersed.


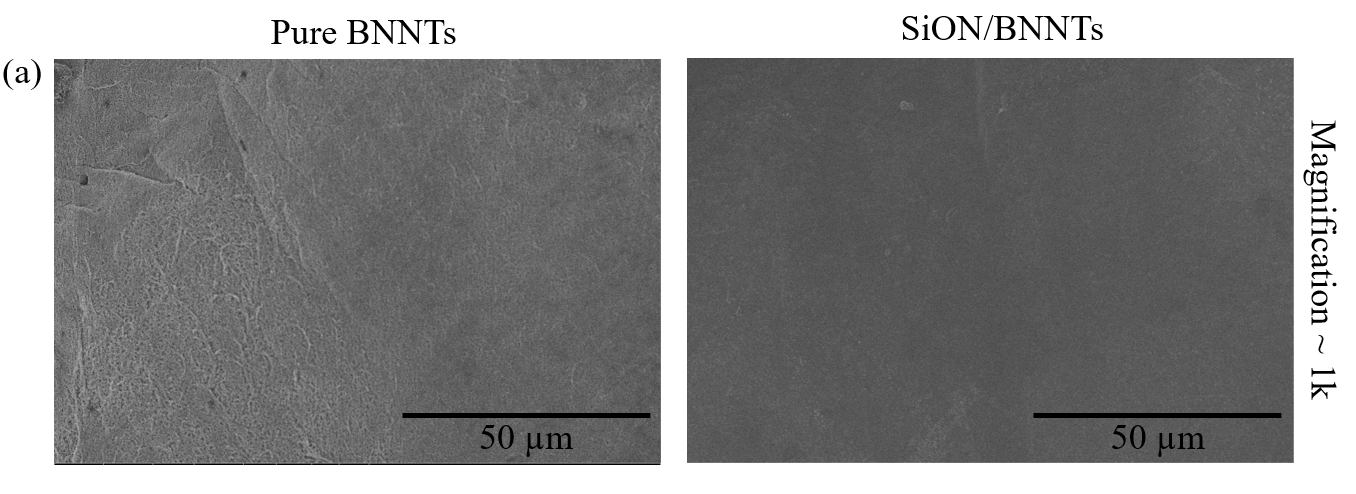


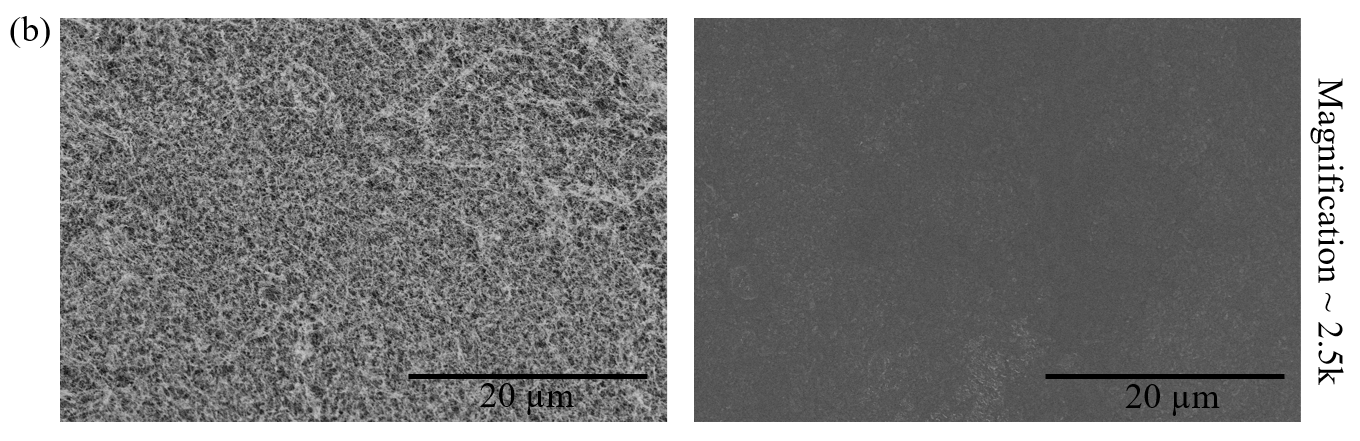


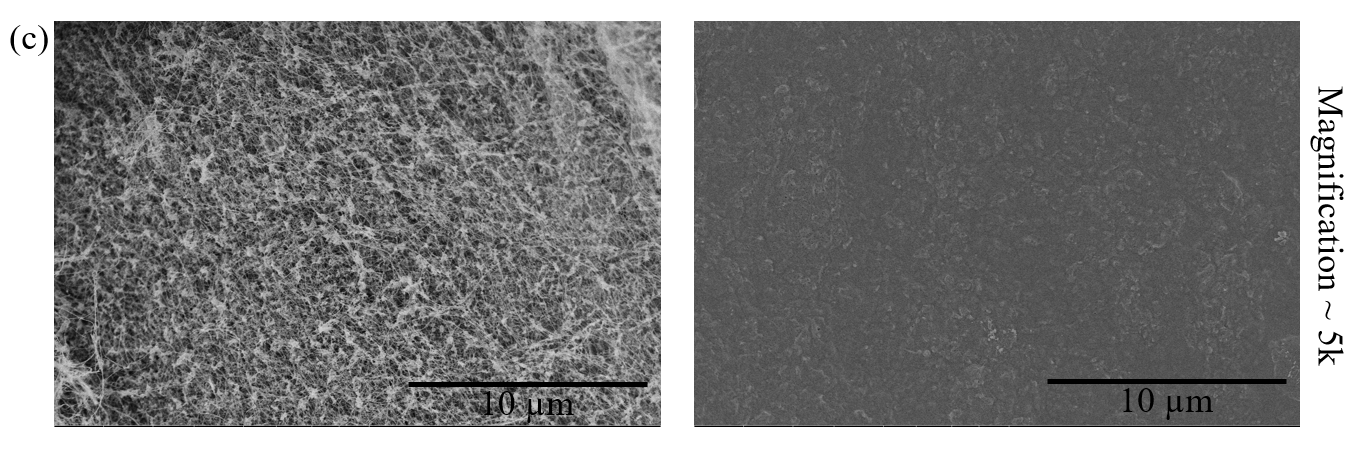


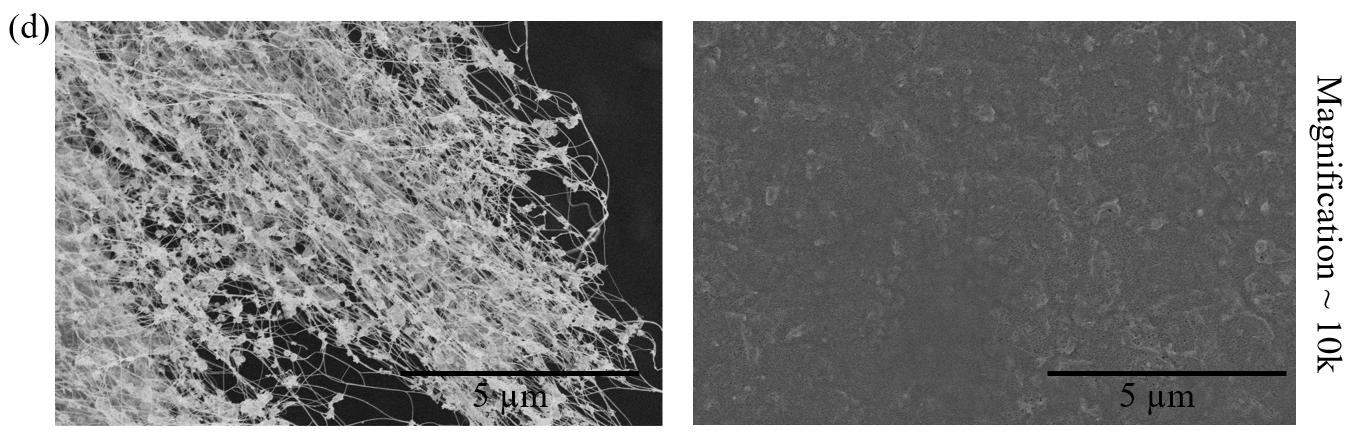


**Fig. S1**. SEM images of (left) pure BNNTs and (right) prepared SiON/BNNTs ceramics, with the magnification of (a) 1k, (b) 2.5k, (c) 5k, and (d) 10k.

1. Cross-sectional SEM image of SiON/BNNTs

It uncovers the true state of the cross-sectional area of our prepared SiON/BNNTs, suggesting that most gaps are indeed fulfilled by SiON. It looks like the BNNTs grow up from the ceramic matrix.


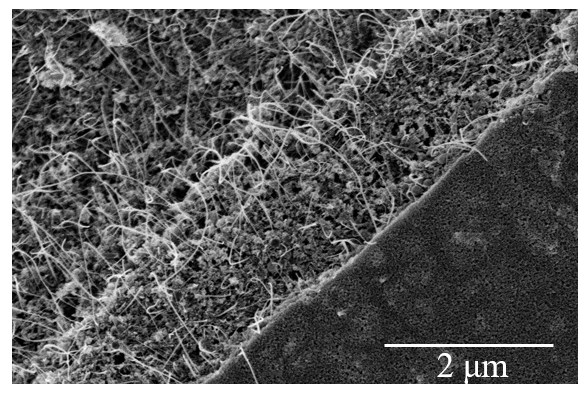


**Fig. S2**. Structural characterization of the cross-sectional area from SiON/BNNTs sample.

1. EDS mapping of BNNTs in SiON

As it can be seen, the analysis indicates a presence of B, Si, O, and N, each with different distributions. The dispersion of BNNTs is clearly illustrated within the SiON matrix.


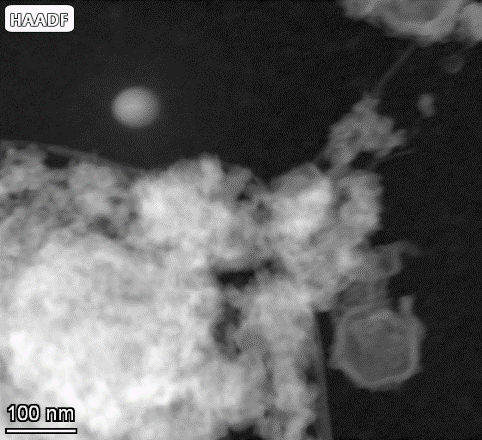

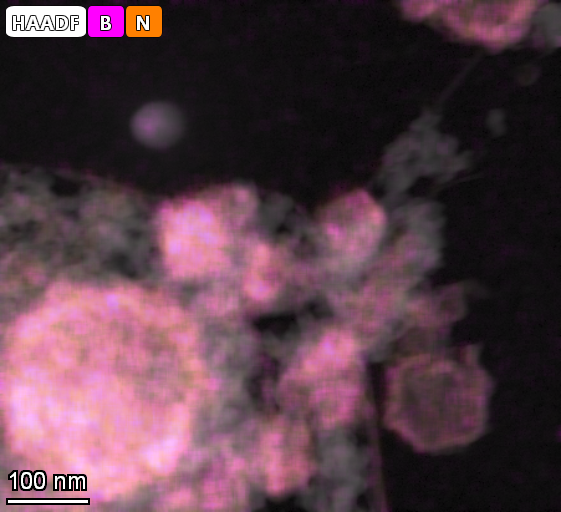

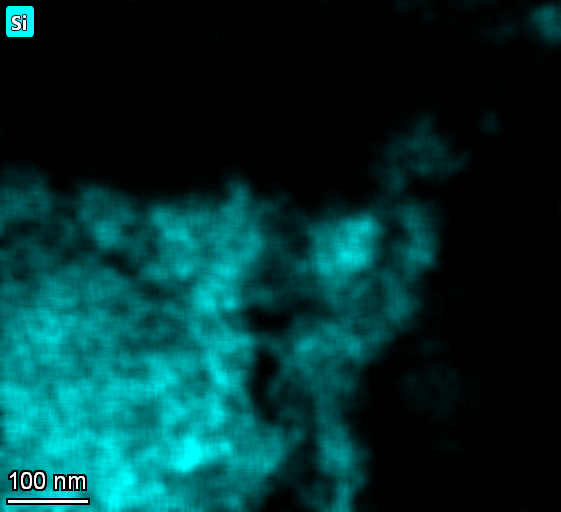

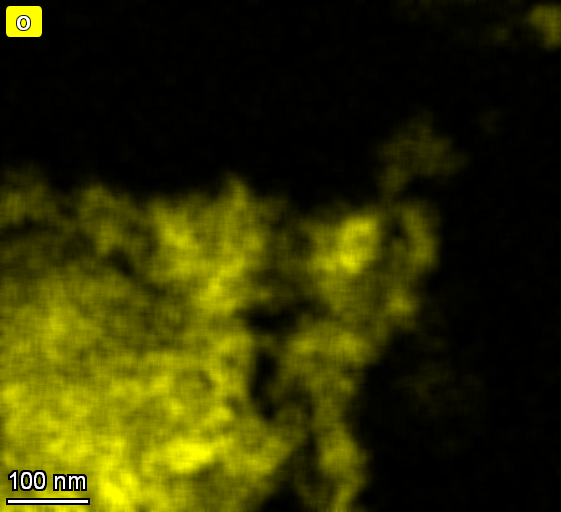


**Fig. S3**. STEM image of the BNNTs/SiON ceramics and related EDS elemental spectrum and maps.

1. Calculation/estimation of weight ratio of BNNTs/SiON

The density of BNNTs mat: 8 mg/cm^2^

Take an area of 25 cm^2^ (5 cm * 5 cm) BNNTs and infiltrate them with PHPS. After drying, the weight of the SiON/BNNTs is: 238 ± 6 mg

Thus,

the mass of BNNTs: 25 * 8 = 200 mg

the mass of SiON: 38 ± 6 mg

the weight ratio of SiON/BNNTs: 0.16 – 0.22
